# Atomic Layer Deposition of Silicon Nitride Thin Films: A Review of Recent Progress, Challenges, and Outlooks

**DOI:** 10.3390/ma9121007

**Published:** 2016-12-12

**Authors:** Xin Meng, Young-Chul Byun, Harrison S. Kim, Joy S. Lee, Antonio T. Lucero, Lanxia Cheng, Jiyoung Kim

**Affiliations:** 1 Department of Electrical Engineering, The University of Texas at Dallas, Richardson, TX 75080, USA; xxm141230@utdallas.edu; 2 Department of Materials Science and Engineering, The University of Texas at Dallas, Richardson, TX 75080, USA; yxb141630@utdallas.edu (Y.-C.B.); hxk161630@utdallas.edu (H.S.K.); jsl160130@utdallas.edu (J.S.L.); atl062000@utdallas.edu (A.T.L.); lxc131530@utdallas.edu (L.C.)

**Keywords:** review, atomic layer deposition, plasma-enhanced ALD (PEALD), silicon nitride, thermal ALD, surface reactions

## Abstract

With the continued miniaturization of devices in the semiconductor industry, atomic layer deposition (ALD) of silicon nitride thin films (SiN_x_) has attracted great interest due to the inherent benefits of this process compared to other silicon nitride thin film deposition techniques. These benefits include not only high conformality and atomic-scale thickness control, but also low deposition temperatures. Over the past 20 years, recognition of the remarkable features of SiN_x_ ALD, reinforced by experimental and theoretical investigations of the underlying surface reaction mechanism, has contributed to the development and widespread use of ALD SiN_x_ thin films in both laboratory studies and industrial applications. Such recognition has spurred ever-increasing opportunities for the applications of the SiN_x_ ALD technique in various arenas. Nevertheless, this technique still faces a number of challenges, which should be addressed through a collaborative effort between academia and industry. It is expected that the SiN_x_ ALD will be further perceived as an indispensable technique for scaling next-generation ultra-large-scale integration (ULSI) technology. In this review, the authors examine the current research progress, challenges and future prospects of the SiN_x_ ALD technique.

## 1. Introduction

Silicon nitride (SiN_x_) has been extensively employed in research and engineering studies. In microelectronics, the SiN_x_ thin films deposited by chemical vapor deposition (CVD) provide many critical functions in the device fabrication process, serving as a dielectric layer, charge storage layer, stress liner, masking layer, barrier, and passivation layer [1,2]. Low-pressure CVD (LPCVD) SiN_x_ films exhibit high conformality and excellent etch resistance [3]. However, the high deposition temperatures (usually ≥700 °C) exceed the thermal budget of advanced ultra-large-scale integration (ULSI) technology. Plasma-enhanced CVD (PECVD) is capable of growing films at low temperatures (≤400 °C) [3]. Unfortunately, the etch resistance and the step coverage performance of PECVD SiN_x_ films usually cannot compete with those of LPCVD SiN_x_ films [4,5]. Moreover, neither of these two techniques can control the film thickness precisely at the atomic-scale due to the intrinsic gas-phase CVD reaction mechanism. In order to deposit high-quality conformal SiN_x_ thin films with a low thermal budget, atomic layer deposition (ALD) is considered a suitable technique [6]. The sequential, self-limiting surface reaction characteristics allow ALD to control SiN_x_ film thickness with atomic-scale precision. The ALD technique has been widely investigated for its performance in depositing thin films for use as high-k dielectrics (e.g., Al_2_O_3_, HfO_2_, ZrO_2_) and interface passivation layers of III−V semiconductors [7,8,9]. For a comprehensive introduction to the ALD technique, readers are referred to many existing reviews on this topic [6,10,11,12,13].

**Figure 1:**
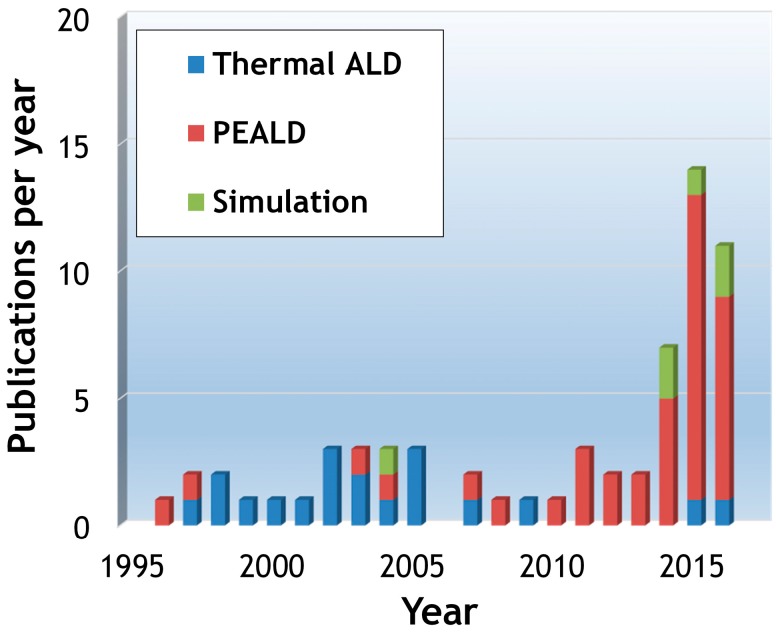
Number of publications per year with respect to atomic layer deposition (ALD) of silicon nitride (SiN_x_) since the first report by Goto et al. was available in 1996 [16], as retrieved in the Web of Science and Google Scholar (through 31 July 2016). The key words for searching included the combination of “atomic layer deposition/atomic layer CVD/plasma-enhanced ALD/plasma-assisted ALD/ALD/PEALD” and “silicon nitride/Si nitride/Si_3_N_4_/SiN/SiN_x_”. Irrelevant publications were not considered.

To date, SiN_x_ thin films have been successfully deposited by both thermal ALD and plasma-enhanced ALD (PEALD). While thermal ALD relies on substrate and chamber heating to drive reaction kinetics at the surface, plasma-enhanced ALD, as the name suggests, takes advantage of the additional energy supplied from plasma to enhance the low-temperature reactivity of species, particularly the nitrogen (N) source, such as ammonia (NH_3_) [14,15]. The need for highly conformal SiN_x_ thin films in the semiconductor industry has driven intense interest in SiN_x_ ALD, namely the low-temperature PEALD technique. Figure 1 illustrates the number of publications involving SiN_x_ ALD over the past two decades. Despite the evident interest in and growing need to understand the process, comprehensive reviews on SiN_x_ ALD are not available. The main goal of this work is to provide a brief yet insightful overview of this technique, discuss the current research progress and describe the challenges of SiN_x_ ALD through existing publications.

The structure of the review has been organized in the following manner. Section 2 will discuss the current research progress, describing first the growth of SiN_x_ thin films via ALD (thermal ALD and PEALD, respectively) and clearly tabulating the key process parameters and results from available reports (through 31 July 2016). Section 2 will also outline the current and potential applications of this technique, highlighting the importance of surface reaction chemistry in SiN_x_ ALD through discussion of several studies. Section 3 will elaborate upon the challenges facing SiN_x_ ALD from several aspects. Finally, Section 4 will conclude this work, providing an outlook of the SiN_x_ ALD technique.

## 2. Current Research Progress

In this section, a number of available reports on ALD SiN_x_ were collected and analyzed for both thin film growth parameters and applications as well as studies on the surface reaction mechanism.

### 2.1. Growth of Silicon Nitride (SiN_x_) Thin Films via Atomic Layer Deposition (ALD)

#### 2.1.1. Thermal ALD

As summarized in Table 1, previous research has demonstrated that growth of SiN_x_ thin films via thermal ALD can be achieved using chlorosilanes as the silicon (Si) source and either ammonia (NH_3_) or a more reactive hydrazine (N_2_H_4_) as the nitrogen (N) source. Thermal ALD SiN_x_ films are typically deposited at high temperatures (above 450 °C). In the pioneering work by Morishita et al. in 1997, ALD SiN_x_ films were successfully deposited using Si_2_Cl_6_ and N_2_H_4_ between 525 and 650 °C [17]. However, hydrazine (N_2_H_4_), commonly used as rocket fuel, is a very dangerous chemical to handle [18]. NH_3_ is considered a good alternative reactant, and has eventually become the most widely used reactant for SiN_x_ thermal ALD. As shown in Table 1, several chlorosilane precursors including SiCl_4_, SiH_2_Cl_2_, Si_2_Cl_6_ and Si_3_Cl_8_ have been extensively investigated for SiN_x_ thermal ALD [17,19,20,21,22,23,24,25,26,27,28,29,30,31,32,33,34]. It is also particularly important to point out the fact that SiN_x_ thermal ALD using non-chlorosilane-based precursors has not yet been reported.

#### 2.1.2. Plasma-Enhanced ALD

Plasma-enhanced ALD (PEALD) is attracting great attention due to its many advantages [14,39]. No longer limited to chlorosilane precursors and NH_3_ plasma, PEALD offers a greater variety of choice for both silicon precursors (SiH_4_, trisilylamine, alkyl-aminosilanes, etc.) and plasma gases (N_2_, N_2_/H_2_). PEALD SiN_x_ films are typically deposited at low temperatures (below 500 °C). As shown in Table 2, an overview of SiN_x_ deposition via PEALD is tabulated.

Direct, remote plasma or radical-assisted types of reactor design allow for versatile process control of ion energy and ion flux density towards the substrate. A more detailed description of the types of reactor designs for PEALD can be found in another review paper [14]. The standard PEALD process only employs plasma during the reactant gas steps and plasma treatment steps. Recently, a report has proposed modifying the process sequence by using an inert gas plasma (such as argon plasma) to activate silicon precursors [40]. It should be noted, however, that any plasma-enhanced mechanism may also incorporate parts of the thermal ALD reaction kinetics, especially at higher temperature.

**Figure 2:**
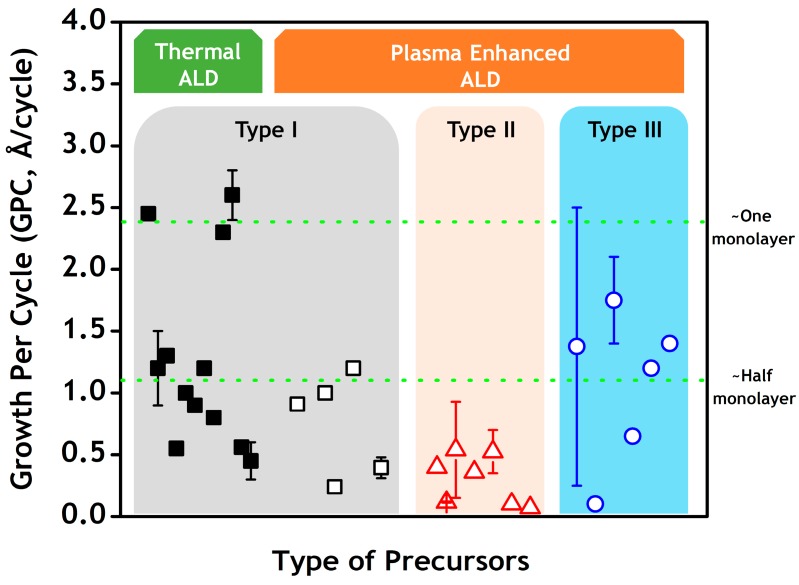
Plot of SiN_x_ ALD growth per cycle (GPC) data (from Table 1 and Table 2) vs. different types of silicon precursors using thermal ALD (solid symbol) and plasma-enhanced ALD (open symbol).

The silicon precursors for SiN_x_ ALD can be simply classified into three general types, as shown in Table 3. Type I precursors are the chlorine-containing precursors (chlorosilanes), which are easy to synthesize, cost-effective, and typically have a good thermal stability. Type II precursors (with carbon) and type III precursors (without carbon) both belong to the chlorine-free subset of precursors. Alkyl-aminosilanes are representatives of type II precursors, which are free of chlorine atoms and demonstrate decent thermal stability. However, these precursors contain carbon atoms, which raises the concern of carbon residue in the films [41]. In addition, a lower growth per cycle (GPC) is typically observed when using a type II precursor. As shown in Figure 2, the GPC is typically less than 1 Å/cycle. This behavior coincides well with the large, complex structure of the molecules. The use of chlorine-free and carbon-free precursors (type III) is considered a promising solution to address impurity concerns. However, the thermal stability and vapor pressure of precursors, the film conformality, and the film quality still need to be carefully evaluated.

Although the first study of SiN_x_ thermal ALD was reported by Morishita et al. in 1997 [17], the first SiN_x_ PEALD process was reported by Goto et al. in 1996, one year earlier [16]. Both of these initial reports used dichlorosilane (SiH_2_Cl_2_) as the Si precursor. Yet, the PEALD process reported growth between 250 and 400 °C using a remote NH_3_ plasma, while the thermal ALD process required a much higher growth temperature (375–500 °C). Following these initial reports, investigations into SiN_x_ ALD continued to focus on type I chlorosilane precursors. Type II and type III precursor studies were not reported until nearly a decade later.

In 2008, Fang et al. demonstrated SiN_x_ ALD using a type II precursor tris(dimethylamino)silane (3DMAS, SiH(N(CH_3_)_2_)_3_) and remote inductively coupled (ICP) N_2_/H_2_ plasma [51]. However, the film exhibited a 5–10 atomic % carbon impurity concentration, which might seriously degrade film quality. Bis(tertiary-butyl-amino)silane (BTBAS, SiH_2_(NH^t^Bu)_2_) is another candidate type II precursor. When employed in an LPCVD SiN_x_ process, BTBAS allows the growth to occur at a reduced temperature regime (550–600 °C) [66]. Recently, Knoops et al. reported a PEALD SiN_x_ process using BTBAS and remote ICP N_2_ plasma [53,54,55,56,67]. High-quality films were obtained (e.g., high mass density ~2.9 g/cm^3^, high R.I. ~1.96, N/Si ratio ~1.4, low H content ~5%, and extremely low wet etch rate ~1 nm/min in 7:1 buffered HF) and a much lower carbon impurity (~2 atomic %) concentration was reported than that from previous studies [51]. The high mass density and low H content could account for the low wet etch rate of the films [52,68,69]. However, the main shortcoming of this novel process was the fairly low GPC (0.32–0.15 Å/cycle at 200–500 °C). The chemical formula of BTBAS is C_8_H_22_N_2_Si, meaning that each of these large molecules contains only one Si atom. Knoops et al. have attributed the low GPC to both the large size of the BTBAS molecule, which could contribute to steric hindrance effects during the growth, and the low density of Si feedstock per precursor molecule.

A concern of both type I and type II precursors is chlorine or carbon impurity incorporation into the film. The use of type III precursors can fundamentally address this concern. King reported a SiN_x_ ALD process using a standard industrial precursor SiH_4_ and direct N_2_ plasma [4,61]. However, a long N_2_ plasma exposure time (>60 s) was required to achieve saturation in GPC using this process because of the short lifetime of the reactive atomic nitrogen species (N, N^+^) [70]. Other chlorine-free and carbon-free precursors such as trisilylamine (TSA, N(SiH_3_)_3_) or neopentasilane (NPS, (SiH_3_)_4_Si) are also considered good candidates for PEALD SiN_x_ [2,5,63,64,65]. Compared to the SiH_4_ molecule, which has only one Si atom, TSA has three Si atoms and one N atom in the molecule while NPS has five Si atoms in the molecule. With a higher building block concentration (Si, N atoms) of the silicon nitride chemical structure in the precursor molecule, these precursors might be helpful in improving GPC [53]. Triyoso et al. have grown SiN_x_ films using TSA and direct N_2_/H_2_ plasma at 300 °C and 400 °C, achieving a reasonably high GPC (1.4–2.1 Å/cycle) and refractive index (R.I.) (2.04–2.16) [5]. Coupled with a different plasma (remote, NH_3_), Jang et al. also successfully demonstrated SiN_x_ ALD using TSA [2,63,64]. Compared with the former study, this process showed a lower GPC (0.65 Å/cycle) and a lower R.I. (1.65–1.80) within a low-temperature regime from 150 to 350 °C. This can likely be attributed to differences between the two deposition systems and the plasma chemistry (direct N_2_/H_2_ plasma vs. remote NH_3_ plasma), which can consequently make differences in the plasma density and the composition of gas-phase nitrogen-containing reactive species. Recently, Weeks et al. compared two type III precursors (NPS and TSA) using direct N_2_ plasma ALD [65]. At identical process conditions, the films grown with NPS had a slightly higher GPC (1.4 Å/cycle vs. 1.2 Å/cycle), though they demonstrated a slightly lower R.I. as described in Table 2.

### 2.2. Applications of SiN_x_ ALD

Due to the decreasing device size and expanding interest in 3D integration, it is indispensable to grow SiN_x_ thin films with superior step coverage and precise thickness control. ALD proves to be effective in meeting the demanding requirements and offers several additional benefits. In this section, applications of the SiN_x_ ALD technique will be discussed in detail.

#### 2.2.1. Gate Spacer

Considerable effort has been devoted to using ALD SiN_x_ thin films as gate spacers in advanced logic and memory devices. A gate spacer acts as a sidewall protection layer of the gate stack and defines the regions of ion implantation. As device dimensions continue downscaling and design rule complexity continues to increase, three requirements for gate spacers should be considered [53]:

- Good conformality
  A gate spacer must be conformal with minimal wafer loading effects, which ensures that spacer thickness is homogeneously distributed across the wafer. Additionally, the spacer shape is a critical factor that will affect the profile of implanted dopants and will, therefore, define the p-n junction. A steeper sidewall will result in a more well-defined source/drain region [5].
- Good etch resistance
  High-k metal gate (HKMG) technology has replaced poly/SiON technology in advanced technology nodes [71,72]. Gate spacers with a low wet etch rate (i.e., good etch resistance) are required to keep the encapsulated HKMG stacks still intact after the subsequent cleaning and etching steps [43].
- Low deposition temperature
  A low deposition temperature can prevent undesirable regrowth of the HKMG stack (e.g., oxidation of TiN, property changes of high-k dielectrics) [43,73,74,75]. In addition, a low process temperature after implantation is beneficial in preventing the diffusion of dopant atoms [2,43].

In an early investigation, Yang et al. reported that using an ALD SiO_2_/SiN_x_ thin film stack as the gate spacer functioned to reduce the short channel effect for sub-90 nm technology [46]. Triyoso and Koehler et al. employed low-temperature (300–500 °C) PEALD SiN_x_ thin films as the spacer or encapsulation liner for 32/28 nm HKMG technology [5,42,43,76]. They found that the transistors using a PEALD SiN_x_ spacer demonstrated better performance than those using a PECVD SiN_x_ spacer. For future 7/5 nm technology nodes, PEALD SiN_x_ can still function as the gate spacer for III–V and Ge high mobility channel transistors [77]. Recently, Djara et al. reported CMOS-compatible n-channel InGaAs on-insulator FinFETs using PEALD SiN_x_ gate spacers [78].

#### 2.2.2. Gate Dielectric

Prior to the prevalence of high-k materials as the gate dielectric, SiN_x_ had been considered an attractive candidate to replace SiO_2_ because of its higher dielectric constant and ability to suppress boron penetration through gate dielectrics [22]. Nakajima et al. demonstrated that using ALD SiN_x_ as the gate dielectric material could significantly suppress boron penetration and improve reliability [20,21,22,23,24,25,26]. Hong et al. reported a SiN_x_(ALD)/SiO_2_/SiN_x_(ALD) sandwich-structure as a tunneling gate dielectric for flash memory application [79,80]. In comparison with a single-layer tunneling dielectric using SiO_2_, the sandwich-structured tunneling dielectric demonstrates a tunable tunneling current with both a higher tunneling current under high electric field and a lower tunneling current under low electric field. These tunneling current-voltage characteristics are attainable due to the modified tunneling barrier profile and are helpful in obtaining better programming characteristics and data retention in flash memory. In addition, ALD SiN_x_ can also serve as a charge trap layer in charge trap flash (CTF) memory and as a part of inter-poly dielectric (IPD) stacks (e.g., oxide-nitride-oxide stacks) in floating gate flash memory [2,63,64].

Gallium nitride metal-insulator-semiconductor high electron mobility transistors (GaN MIS-HEMTs) are being studied for power device applications [7,81,82,83,84,85,86,87,88]. However, it has been reported that trapping at the oxide dielectric/GaN interface, which might originate from Ga–O bonds, may contribute to instability in the threshold voltage (V_th_) [89,90]. A nitride-based gate dielectric such as SiN_x_ can potentially improve the interface quality [91,92]. Recently, several researchers employed PEALD SiN_x_ as a gate dielectric layer of GaN MIS-HEMTs and achieved improvement of power device performance [93,94,95,96,97,98,99,100]. This suggests that the application of ALD SiN_x_ could extend to GaN power electronics.

#### 2.2.3. Encapsulation Layer

Encapsulation of flexible organic devices is widely adopted to isolate devices from ambient moisture and oxygen. Low process temperatures (generally <120 °C) are required to avoid thermal degradation of the organic layers [101,102,103]. Employing an inorganic ALD film (e.g., Al_2_O_3_, SiO_2_) as a moisture permeation barrier layer has been considered a good approach [103,104,105,106,107]. However, such low temperatures are often not viable for SiN_x_ thermal ALD because the thermal energy is insufficient. In 2011, King showed that PEALD SiN_x_ films deposited using SiH_4_ and N_2_ plasma had excellent moisture-barrier performance [4]. However, the relatively high deposition temperature (250–400 °C) is not compatible with organic devices. More recently, Andringa et al. found that outstanding moisture-barrier performance could be obtained using PEALD SiN_x_ deposited at a low temperature (80–200 °C) [55].

#### 2.2.4. Deposition of Composite Films

Another benefit of ALD is the so-called “digital alloying” or “digital doping” capability. It is a unique and facile route for obtaining ternary or quaternary composite thin films. Many composite materials including III–N semiconductors (Al_x_Ga_1−x_N, In_x_Ga_1−x_N, etc.) and oxides (Hf_x_Zr_1−x_O_2_, Al-doped ZnO, etc.) have been prepared with this kind of approach [108,109,110,111,112,113]. Based on this concept, Kim et al. successfully reduced the dielectric constant of ALD SiN_x_ gate spacer by alloying with boron nitride [114]. They demonstrate that this technique can reduce parasitic capacitance. Recently, PEALD SiN–AlN composite films with excellent etch resistance in HF acid were also developed by Kim et al. [52]. In addition, researchers also studied ALD–TiSiN for use as a gate electrode and diffusion barrier, and ALD–RuSiN composite films for use as a Cu diffusion barrier [58,59,62,115,116,117,118].

#### 2.2.5. Stressor

The channel strain can be intentionally modulated to enhance the carrier mobility by utilizing stressor layers [119]. PECVD SiN_x_ films with high tensile/compressive stress have been used as contact etch stop liners (CESL) and adopted in stress memory technique (SMT) to improve CMOS transistor performance [120,121]. However, reports regarding how to control the stress in ALD SiN_x_ thin films are scarce. Nagata et al. found that the stress of PEALD SiN_x_ film was dependent on the deposition temperature [47]. PEALD SiN_x_ films showed higher stress and better stress uniformity, in comparison with the SiN_x_ films deposited by thermal ALD. However, other important factors (e.g., precursors, plasma conditions, plasma treatment) which may modulate the stress were not studied. King and Triyoso et al. reported that the intrinsic stress of PEALD SiN_x_ films could be modulated to be either compressive or tensile by varying low-frequency power [4,5], which also holds true for PECVD SiN_x_ films [122,123].

### 2.3. A Highlighted Topic: The Surface Reactions of SiN_x_ ALD

**Figure 3:**
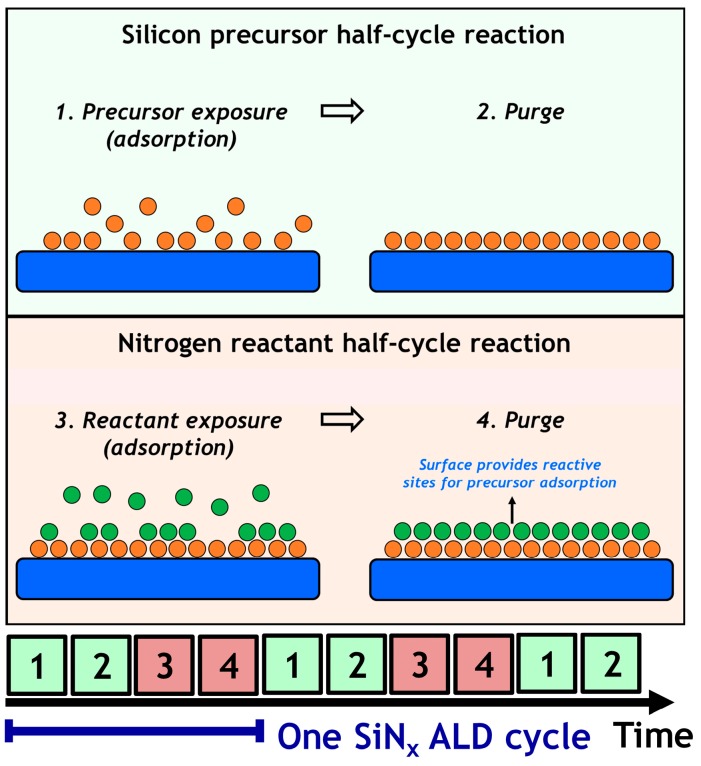
Schematic representation of one SiN_x_ ALD cycle.

It is well known that ALD is based on the self-limiting surface reactions. Thus it is essential to understand the surface reaction mechanism. SiN_x_ ALD consists of two half-cycle reactions: “Silicon precursor half-cycle reaction” and “Nitrogen reactant half-cycle reaction”, as shown in Figure 3.

During the silicon precursor half-cycle reaction, chemisorption interactions allow precursor molecules to “stick” to the surface. Excess precursor molecules which are physisorbed and reaction byproducts will be removed during the purging step. Consequently, the new surface is dominantly terminated by ligands from the adsorbed silicon precursor molecules (e.g., alkylamino groups, –SiCl_x_ groups). The nitrogen reactant half-cycle reaction serves the purpose of eliminating the undesired surface components (such as the ligands containing carbon, chlorine, hydrogen) and forming the silicon-nitrogen bonds. Eventually, after one cycle reaction, the dominant components on the new surface will be nitrogen-containing reactive sites (e.g., under-coordinated N atoms, –NH_x_, dangling bonds). As recently elaborated in the paper authored by Ande et al., the relation between the two half-cycle reactions of SiN_x_ ALD should be reciprocal: the surface after the former reaction can facilitate the following reaction, otherwise, the film growth will not be sustainable [49].

Experimentally, the surface reactions have been investigated using in situ Fourier Transform Infrared Spectroscopy (FTIR), Optical Emission Spectroscopy (OES), and Quadrupole Mass Spectrometry (QMS) [19,27,32,33,56,67]. For example, Klaus et al. analyzed the surface FTIR spectrum for the thermal ALD reaction between SiCl_4_ and NH_3_ [19]. The repeated recurrence of amino and chlorine characteristic peak revealed the reconstruction of surface components after each half-cycle reaction. In addition to the FTIR analysis on the film surface, Bosch et al. recently combined FTIR, OES, and QMS to analyze the gas-phase species during the ALD reactions between BTBAS and N_2_ plasma [56,67]. This systematic study has revealed a lot of useful information such as surface termination, plasma species, impurities in the films, and reaction byproducts.

Calculations based on first-principles Density Functional Theory (DFT) are widely employed to determine thermodynamics and kinetics for ALD SiN_x_ reactions. The early stage studies focused on the adsorption of an individual molecule such as SiH_4_, chlorosilane or NH_3_ on the silicon surface and did not specifically focus on ALD reactions [124,125]. These early works preceded systematic studies of SiN_x_ ALD surface reactions and formed the cornerstone for later theories. Later in 2004, Mui et al. investigated SiN_x_ ALD with SiH_4_ and NH_3_ using DFT calculations [126]. This pioneering work has revealed that the reactions have to overcome high activation barriers which is challenging for thermal ALD [127]. Recently, the collaboration between academia and industry has contributed to the surge of theoretical simulation studies of SiN_x_ ALD [34,54,57,127,128,129]. Murray et al. found that the orientation of hydrogen atoms connected to the hydroxyl (–OH) and amine (–NH_2_) groups is quite different [128]. To form Si–N bonds, the silicon precursor molecules have to interact with amine groups laterally. This pathway is more difficult than the interactions with hydroxyl groups in a vertical way to form Si–O bonds. In terms of aminosilane molecules, Huang et al. predicted the high-activation barriers of the ALD half-cycle reactions between bis(diethylamino)silane (BDEAS, C_8_H_22_N_2_Si), bis(tertiary-butyl-amino)silane (BTBAS, SiH_2_(NH^t^Bu)_2_) and amine groups [127]. Interestingly, the above predictions yielded good agreement with the experiment results reported by Knoops et al., who demonstrated high-quality ALD SiN_x_ thin films using BTBAS and N_2_ plasma [53]. In contrast, the attempts to deposit SiN_x_ using N_2_/H_2_ or NH_3_ plasma failed. Both the experimental results and DFT calculation reported by Ande et al. reveal that the effective adsorption of silicon precursor molecules (BTBAS) only occurs on a surface composed of under-coordinated nitrogen and silicon atoms [54]. The interactions between silicon precursor molecules and under-coordinated atoms are favorable as long as the steric hindrance effects of nearby –NH_x_ and –H surface groups are not present. An N_2_ plasma, without the presence of hydrogen-containing reactive species (e.g., NH_x_, H), can create a surface composed of under-coordinated nitrogen and silicon atoms. In contrast, a hydrogen-containing plasma (H_2_, N_2_/H_2_, or NH_3_ plasma) can passivate the under-coordinated nitrogen and silicon atoms by generating –NH_x_ and –H surface groups. The presence of these surface groups is unfavorable for the adsorption of silicon precursor molecules.

Similar findings were also reported by King and Yusup et al., investigating SiN_x_ ALD using SiH_4_ and chlorosilanes (SiCl_4_ and Si_2_Cl_6_) as the respective silicon precursor [4,34]. It is necessary to point out the fact that an NH_3_ gas or NH_3_ plasma exposure step is still essential when using chlorosilane as the silicon precursor. The nitrogen radicals in an N_2_ plasma are not reactive with the chlorine ligands. Nevertheless, Yusup et al. have demonstrated that introducing an additional N_2_ plasma treatment step prior to Si_2_Cl_6_ exposure in a thermal ALD cycle (process sequence: purge/Si_2_Cl_6_/purge/NH_3_ gas/purge/N_2_ plasma treatment) can increase the GPC nearly twice (from 0.59 Å/cycle to 1.1 Å/cycle) [34]. The Si_2_Cl_6_ dosage for the saturation growth has also been reduced by approximately one order of magnitude (from 10^7^ L to <10^6^ L). The authors have proposed that an N_2_ plasma is able to recover the under-coordinated surface atoms and consequently enhance the adsorption of Si_2_Cl_6_ molecules.

## 3. Challenges of SiN_x_ ALD

While SiN_x_ deposition via ALD offers several benefits over LPCVD and PECVD approaches, many challenges including the inherent slowness of film growth still exist and hinder the industrialization of SiN_x_ ALD. In this section, we will discuss several critical challenges with SiN_x_ ALD.

### 3.1. Thermal ALD

#### 3.1.1. Limitation of the Deposition Temperature

From the perspectives of process integration, the high temperature of LPCVD process (usually ≥700 °C) can be avoided by using a thermal ALD process. As mentioned in Section 2, because of the high-energy barriers of each half-cycle ALD reaction, the deposition temperatures of SiN_x_ thermal ALD typically exceed 450 °C. To obtain sufficient reactivity and desired film quality, thermal ALD SiN_x_ still requires a higher deposition temperature (500–600 °C). The processes within this temperature regime may not be compatible with the advanced ULSI technology and the chlorosilane precursors may be subjected to thermal decomposition [32]. Consequently, thermal ALD of high-quality SiN_x_ films can only be applied to the applications without a rigid restriction on high deposition temperature. Therefore, it is necessary to employ the extra energy supplied from plasma or other pathways to reduce the deposition temperature without sacrificing the SiN_x_ film quality.

#### 3.1.2. Excess Precursor Dosage and Dosing Time

Chipmakers have always been sparing no efforts to maximize the throughput (e.g., reduce process time per wafer) and minimize the cost (e.g., reduce material consumption). In terms of the ALD process, once the saturation growth is satisfied, minimizing the dosage and the dosing time of precursor is desired. However, the previous reports (see Table 4) showed that a high dosage of precursor (10^7^–10^10^ L) was required for the saturated reaction in SiN_x_ thermal ALD. The dosing pressure of precursor is typically in the range of 0.1–10 Torr, which corresponds to approximately 10^1^–10^3^ s dosing time. This raises great challenges from the manufacturing point of view. Fortunately, as also shown in Table 4, the use of plasma-enhanced ALD or hot filament (HF) ALD could greatly reduce the precursor dosage by several orders of magnitude [16,29,44,49].

### 3.2. Plasma-Enhanced ALD

#### Degradation of the Conformality

**Figure 4:**
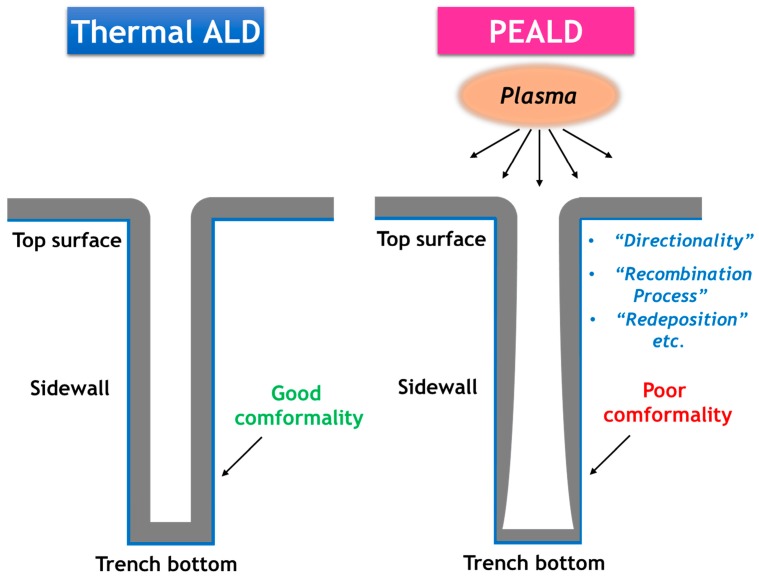
Schematic diagram of conformality degradation in PEALD, in comparison with thermal ALD.

Due to the superior step coverage of thermal ALD, it has become the method of choice for depositing SiN_x_ thin films conformally on high aspect ratio (AR) structures. However, as shown in Figure 4, this unique feature which differentiates thermal ALD from the other methods becomes less straightforward when plasma is involved [65]. The directionality and short mean free path of reactive species in the plasma become detrimental for high AR and complexed features. For example, a typical trench structure consists of the top-surface, the sidewall, and the trench bottom. The transformation of reactive radicals in the nitrogen-containing plasma to non-reactive molecules (e.g., N_2_, H_2_) will occur, through a “recombination process” proposed by Knoops et al. [130]. Consequently, different reactivity and growth rates will occur selectively at different locations, resulting in the degraded step coverage and the variation of film properties (density, composition, wet etch rate, etc.) throughout the structure. In addition, Knoops et al. found that the byproduct components in the gas phase could be unintentionally “redeposited” back to the film surface [56,67]. Thus, the effects of directionality, “recombination process,” and “redeposition” should be carefully reduced by optimizing the plasma conditions [14].

In addition, the selection of silicon precursor and nitrogen-containing plasma gas is crucial for improving conformality [131,132,133]. For example, Tang et al. have reported that the conformality of an N_2_-plasma-based process is inferior to that of an NH_3_-plasma-based process [131,132]. This can be attributed to the fact that the lifetime of reactive radicals in an N_2_ plasma is shorter than that in an NH_3_ plasma. Additionally, as explained previously in Section 2.3, the difference in surface termination after plasma exposure (e.g., under-coordinated surface atoms after N_2_ plasma exposure vs. –NH_x_ and –H groups passivated surface after NH_3_ plasma exposure) can influence the adsorption of silicon precursor molecules. This may account for the difference in conformality as well. Nevertheless, the use of N_2_ plasma to grow SiN_x_ films can suppress the hydrogen incorporation directly from the reactive species in the plasma. A lower hydrogen content in the PEALD SiN_x_ films is helpful in reducing the wet etch rate [52,68,69]. It is also beneficial for the applications that prefer a hydrogen-free or ammonia-free plasma process.

### 3.3. Other Common Challenges

#### 3.3.1. Chlorine Impurities and Byproducts from the Chlorine-Containing Precursors

The concern with the high cost of SiN_x_ ALD precursors may be solved by using chlorosilane-based precursors. However, the chlorine impurities and process byproducts, ammonia chloride (NH_4_Cl), raise new challenges [134]. The unwanted incorporation of chlorine may cause a device-reliability issue. Further decreasing deposition temperature below the thermal decomposition temperature (~330 °C) of NH_4_Cl will lead to incomplete removal of byproducts from the surface. Additionally, the defects and undesired system downtime associated with NH_4_Cl will reduce productivity. More importantly, some applications prefer a halide-free process [4,135,136,137].

#### 3.3.2. Substrate Sensitivity

The initial surface reactions of SiN_x_ ALD is extremely critical and immensely sensitive to the substrate that is used [13]. Various materials, including silicon, compound semiconductors, organics, graphite, and two-dimensional (2D) materials (graphene, MoS_2_, black phosphorus, etc.), can be used as the substrate for SiN_x_ ALD [138,139,140,141,142]. This uncertainty in the reaction surface exacerbates the difficulty in creating a universal SiN_x_ ALD process to fulfill all requirements. For example, Yokoyama et al. observed that there was a growth delay in ALD SiN_x_ on the LPCVD Si_3_N_4_ surface while no delay was observed on the hydrogen-terminated Si surface [29].

In the past few years, some novel devices based on 2D materials have been proposed for beyond CMOS nanoelectronics [143,144,145,146]. To grow high-k gate dielectrics on 2D materials using ALD, several surface functionalization techniques have been successfully adopted to activate the chemically inert surface [147,148,149]. PECVD SiN_x_ film has been successfully employed as a gate dielectric layer for graphene field-effect transistors (GFET), a passivation layer of MoS_2_ field-effect transistors [139,140,150]. ALD is expected to be a better approach to growing high-quality SiN_x_ thin films on 2D materials. However, the lack of surface reaction sites on 2D materials makes SiN_x_ ALD difficult. To our best knowledge, studies on this topic have not been reported yet. Therefore, SiN_x_ ALD on 2D materials is expected to encounter many technical issues (e.g., non-uniform film growth, unintentional damage, and doping of 2D materials) in the early development stage [151].

## 4. Conclusions and Outlooks

Atomic layer deposition (ALD) is an ideal approach to growing SiN_x_ thin films to fulfill the requirements of ULSI technology downscaling to the sub-10 nm technology node. In the past two decades, there has been remarkable progress in the development of the silicon nitride (SiN_x_) ALD technique.

This review has presented a brief introduction to the motivation of SiN_x_ ALD. Subsequently, SiN_x_ thin film growth using thermal ALD and plasma-enhanced ALD has been tabulated and described in detail. By analyzing the correlation between precursors and growth per cycle (GPC), it is found that the carbon-containing precursors show a lower GPC, which may relate to the size and complexity of the molecule structure. In addition, various critical applications have been examined in depth. Specifically, the importance of surface reactions of SiN_x_ ALD has been discussed. The experimental and theoretical findings will provide the readers with a guideline for the process design and optimization. Finally, some of the challenges faced when working with SiN_x_ ALD have been considered.

Regarding the important applications in semiconductor technology and relatively short development history (~20 years), the prospects for SiN_x_ ALD are very promising as manufacturing approaches atomic-scale dimensions. The design, development and optimization of precursors, process sequences, reactors and plasma sources require collaborative work between the scientific and industrial communities. New findings will be reported to understand the underlying mechanism, and new approaches will be proposed to address the challenges. Additionally, with the improvements of this technique, the applications will be ultimately expanded into those unexploited areas such as biology and the medical industry. It is evident that, shortly, great benefits will be gained from SiN_x_ ALD.

## Figures and Tables

**Table 1:** Overview of silicon nitride thin films deposited by thermal ALD.

Precursor	Reactant	Deposition Temp. (°C)	GPC (Å/Cycle)	Refractive Index	References
SiCl_4_	NH_3_	427–627	2.45	2.01	[19]
SiCl_4_	NH_3_	375, 550–600	0.9–1.5	–	[20,21,22,23,24,25,26]
SiCl_4_	NH_3_	500	~1.3	–	[27]
SiCl_4_	NH_3_	350–400	0.55 *	–	[28]
SiH_2_Cl_2_	NH_3_^(HF)^	375	~1	1.9	[29]
SiH_2_Cl_2_	NH_3_	375, 550	~0.9	–	[30]
SiH_2_Cl_2_	NH_3_	500	~1.2	–	[27]
SiH_2_Cl_2_	NH_3_	450	0.8	~2.3	[31]
Si_2_Cl_6_	N_2_H_4_	525–650	2.3	2.07	[17]
Si_2_Cl_6_	NH_3_	515–557	2.4–2.8	1.7–1.8	[32]
Si_2_Cl_6_	NH_3_	300	0.56	–	[34]
Si_3_Cl_8_	NH_3_	300–500	0.3–0.6	–	[33]

The precursor, the reactant, deposition temperature (°C), growth per cycle (GPC, Å/cycle), refractive index (R.I.) and references are given for reports through 31 July 2016. “HF” is hot filament, which can efficiently dissociate the reactant molecules (e.g., NH_3_) and enhance the reactivity. Readers can find more descriptions of the hot filament chemical vapor deposition (CVD) technique (also known as catalytic CVD or hot wire CVD technique) in the references [35,36,37,38]. “*” is the result of depositing SiN_x_ film on Ge wafer. “–” is not specified.

**Table 2:** Overview of silicon nitride thin films deposited by plasma-enhanced ALD.

Precursor	Plasma Gas	Reactor/Type	Deposition Temp. (°C)	GPC (Å/Cycle)	Refractive Index	References
SiH_3_Cl	NH_3_	Radical/–	400	–	–	[42,43]
SiH_2_Cl_2_	NH_3_	Remote/MW	250–400	0.91	1.6	[16,44]
SiH_2_Cl_2_	NH_3_	Remote/–	350–400	~1.0	–	[45]
SiH_2_Cl_2_	NH_3_	Remote/CCP	595	–	–	[46]
SiH_2_Cl_2_	NH_3_	Radical/–	500	–	–	[42,43]
SiH_2_Cl_2_	NH_3_	–/–	350–500	–	–	[47]
SiH_2_Cl_2_	NH_3_	Remote/ICP	350	0.24	–	[48]
Si_2_Cl_6_	NH_3_	Direct/CCP	350–450	1.2	1.9	[49]
Si_2_Cl_6_	NH_3_	–/–	200–500	0.31–0.38	–	[50]
SiH(N(CH_3_)_2_)_3_	N_2_/H_2_	Remote/ICP	350	0.4	1.95	[51]
SiH(N(CH_3_)_2_)_3_	N_2_	Remote/ICP	350	0.11–0.12	–	[48,52]
SiH_2_(NH^t^Bu)_2_	N_2_	Remote/ICP	100–500	0.15–0.93	1.63–1.96	[53,54,55,56]
C_9_H_29_N_3_Si_3_	N_2_	Direct/CCP	250–400	0.36	1.93	[57]
C_6_H_17_NSi	NH_3_	Direct/CCP	325	0.35–0.7	1.7–1.8	[40]
C_9_H_25_N_3_Si	NH_3_	Direct/CCP	325	<0.1	–	[40]
C_9_H_25_N_3_Si	NH_3_	–/–	270	~0.07	–	[58,59]
C_8_H_22_N_2_Si	N_2_/H_2_	Remote/ICP	–	–	–	[60]
SiH_4_	N_2_	Direct/CCP	250–400	0.25–2.5	1.7–1.85	[4,61]
SiH_4_	N_2_/H_2_	Direct/CCP	350	0.1	–	[62]
(SiH_3_)_3_N	N_2_/H_2_	Direct/–	300–400	1.4–2.1	2.04–2.16	[5]
(SiH_3_)_3_N	NH_3_	Remote/ICP	150–350	0.65	1.65–1.80	[2,63,64]
(SiH_3_)_3_N	N_2_	Direct/CCP	250–300	1.2	2.07–2.2	[65]
(SiH_3_)_4_Si	N_2_	Direct/CCP	250–300	1.4	2.07–2.14	[65]

The precursor, the plasma gas (only nitrogen-containing reactant gas, not carrier gas), reactor type (radical, remote or direct, CCP = Capacitively Coupled Plasma, ICP = Inductively Coupled Plasma, MW = Microwave), deposition temperature (°C), growth per cycle (GPC, Å/cycle), refractive index (R.I.) and references are given for reports through 31 July 2016. “^t^Bu” is tertiary butyl. “–” is not specified. SiH(N(CH_3_)_2_)_3_ = 3DMAS, Tris(dimethylamino)silane; SiH_2_(NH^t^Bu)_2_ = BTBAS, Bis(tertiary-butyl-amino)silane; C_9_H_29_N_3_Si_3_ = DTDN-2H2, DNF Co., Ltd.; C_6_H_17_NSi = DIPAS, Di(isopropylamino)silane; C_9_H_25_N_3_Si = TIPAS, Tris(isopropylamino)silane; C_8_H_22_N_2_Si = BDEAS, Bis(diethylamino)silane; (SiH_3_)_4_Si = NPS, Neopentasilane; N(SiH_3_)_3_ = TSA, Trisilylamine.

**Table 3:** Classification of the silicon precursors used in SiN_x_ ALD process.

Type	Classification	Examples	Major Potential Impurities	Deposition Method
I	Chlorine-containing precursors	Chlorosilanes: SiH_2_Cl_2_, Si_2_Cl_6_, etc.	Cl, H, O	PEALD, Thermal ALD
II	Carbon-containing precursors	Alkyl-aminosilanes: 3DMAS (SiH(N(CH_3_)_2_)_3_), BTBAS (SiH_2_(NH^t^Bu)_2_), etc.	C, H, O	PEALD
III	Chlorine-free and carbon-free precursors	SiH_4_, TSA (N(SiH_3_)_3_), NPS ((SiH_3_)_4_Si), etc.	H, O	PEALD

**Table 4:** Examples of the precursor dosage employed in SiN_x_ ALD for the saturation growth.

ALD	Precursor	Reactant	Dosing Pressure (Torr)	Dosage (L)	Deposition Temp. (°C)	GPC (Å/Cycle)	References
Thermal	SiCl_4_	NH_3_	10	~1 × 10^10^	427–627	2.45	[19]
Thermal	SiCl_4_	NH_3_	170	~5 × 10^10^	375, 550	~0.8	[24]
Thermal	SiH_2_Cl_2_	NH_3_	–	~6 × 10^9^	450	0.8	[31]
Thermal	Si_2_Cl_6_	NH_3_	1	~1 × 10^7^	525–650	2.3	[17]
Thermal	Si_2_Cl_6_	N_2_H_4_	1	~2 × 10^8^	515–557	2.4–2.8	[32]
Thermal	SiH_2_Cl_2_	NH_3_^(HF)^	0.06	~5.4 × 10^6^	375	~1	[29]
Plasma	SiH_2_Cl_2_	NH_3_	0.06	~5.4 × 10^6^	250–400	~0.9	[16,44]
Plasma	Si_2_Cl_6_	NH_3_	~0.07	~8 × 10^5^	350–450	1.2	[49]
Plasma	(SiH_3_)_3_N	NH_3_	0.3	~6 × 10^4^	150–350	0.65	[2,63,64]

Note: Dosage unit “L” represents langmuir, 1 L corresponds to an exposure of 1 × 10^−6^ Torr during 1 s. “HF”: hot filament
